# Toward the Development of a Novel Newborn Screening Modality: In-Depth Nontargeted Proteome Analysis of Dried Blood Spots with a Robotic Pipeline Using Low-Cost Iron Powders

**DOI:** 10.1021/acs.analchem.5c01720

**Published:** 2025-08-12

**Authors:** Daisuke Nakajima, Masaki Ishikawa, Ryo Konno, Yusei Okuda, Hideo Sasai, Osamu Ohara, Yusuke Kawashima

**Affiliations:** Department of Applied Genomics, 34833 Kazusa DNA Research Institute, 2-5-23 Kazusa Kamatari, Kisarazu, Chiba 292-0818, Japan

## Abstract

We developed a simple protein extraction method for dried blood spots (DBS) that potentially meets the throughput required for newborn screening (NBS) and optimizes nontargeted proteomic analysis in combination with liquid chromatography coupled mass spectrometry in the data-independent-acquisition mode (DIA–LC–MS/MS). The developed pipeline, termed **N**on-targeted **A**nalysis of **N**on-specifically **D**BS-**A**bsorbed proteins (NANDA), successfully addressed the following three challenges: (1) processing of 96 3.2 mm DBS punches in parallel using low-cost iron powders with a robotic system, (2) identifying more than 5,000 proteins using DIA–LC–MS/MS, and (3) improving DIA–LC–MS/MS throughput to 45 samples/day with minimal compromise in protein coverage depth. The results imply that this pipeline can open new venues for conducting NBS using nontargeted quantitative proteome profiling, which has been a missing modality in NBS.

## Graphical abstract

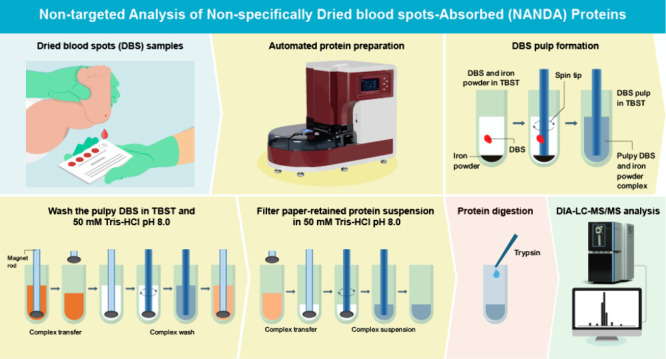

## Introduction

Blood components are an important source of biomarkers for disease detection because they are strictly kept within a normal range by homeostasis in the healthy state. Many diagnostic tools using fresh whole blood, serum, or plasma have been widely utilized. As a modified approach, dried blood spots (DBS) have been used as an alternative to fresh blood because of the simplicity of shipping and storage.^1–4^ For example, DBS have served as a versatile format for newborn screening (NBS) to prevent disease onset and/or reduce symptoms by early intervention. The DBS approach is well-established and has proven effective in conventional NBS based on the measurements of specific metabolites or enzymatic activities.^5^ As the number of treatable diseases increases with the emergence of new treatments, new NBS modalities have been introduced. Examples include measurement of the *SMN1* gene copy number and T/B-cell excision circle DNA for spinal muscular atrophy^6^ and severe combined immunodeficiency,^7^ respectively. However, an increase in screening items inevitably leads to increased screening costs and labor, presenting a serious concern. To address this issue, we are actively seeking an appropriate “one-size-fits-all” screening modality. To this end, we propose measuring biomolecular profiles in a nontargeted manner, followed by data filtering, as a solution. Similarly, nontargeted genome sequencing analysis has attracted attention as a new NBS modality. This nontargeted approach could enable us to conduct NBS for multiple diseases using a “one-size-fits-all” platform. However, although genome sequencing may serve as a versatile screening approach for various inherited diseases, various debates regarding its suitability for NBS, including ethical, economic, and social issues, are currently underway. Additionally, a concern arises that genomic information, which pertains to genotype, does not fully convey the molecular phenotype of neonates, on which current NBS is based before clinical symptoms appear. In this context, we propose nontargeted quantitative protein profiling as a novel NBS modality, offering an alternative to genome sequencing. In a proof-of-concept study, we recently demonstrated that a nontargeted proteomic approach using data-independent acquisition liquid chromatography–tandem mass spectrometry (DIA–LC–MS/MS) on DBS could offer complementary insights into personal genome sequences for diagnosing inborn errors of immunity.^8^ However, the pipeline was labor-intensive, and the overall throughput was not high enough for NBS, suggesting that a modified pipeline for the nontargeted proteome analysis of DBS must be developed to move beyond the proof-of-concept stage.

In this study, we thus set the following goals to be achieved by a new pipeline: 1) capability of the parallel processing of 96 DBS punches using automation at low cost, 2) deeper DBS proteome coverage than that in our previous method, and 3) improvement of overall throughput to 10,000 samples/year/LC–MS instrument. By introducing several new methodologies, we successfully constructed a new pipeline that robotically extracts proteins from 96 individual DBS punches in parallel with a protein coverage of 4,858 at a throughput of 45 DBSs/day. We believe that the developed DBS-manipulation pipeline paves the way for introducing nontargeted protein profiling as a new modality in NBS.

## Materials and Methods

### DBS Preparation

DBSs were acquired from consenting healthy volunteers via a finger prick onto blood sampling papers (ADVANTEC PKU-S; ADVANTEC; Whatman 903 Protein Saver Cards, Whatman; PerkinElmer 226 Spot Saver Card; PerkinElmer), which were dried overnight.

### Protein Preparation Method

A flowchart showing the preparation of proteins for the **N**on-targeted **A**nalysis of Non-specifically **D**BS-**A**bsorbed proteins (NANDA) is provided in Figure 1. A 3.2 mm-diameter disk punched out from a DBS was added to 500 μL of tris buffered saline with Tween 20 (TBST), crushed into a pulpy DBS with vigorous agitation for 30 min at maximum using a stirring centrifuge (NSD-12; Nissin Rika Co., Ltd., Tokyo, Japan), and centrifuged for 5 min at 15,000 × *g*. Thereafter, the supernatant was removed, and pulpy DBS was isolated (Step 1,2). Pulpy DBS was washed by adding 500 μL of TBST, agitated vigorously for 10 min at maximum using a stirring centrifuge, and centrifuged for 5 min at 15,000 × *g*, after which the supernatant was removed (Step 3). Next, 500 μL of 50 mM Tris-HCl at pH 8.0 was added, and the mixture was agitated vigorously for 10 min at maximum using a stirring centrifuge and centrifuged for 2 min at 15,000 × *g*, followed by supernatant removal. Afterward, another 500 μL of 50 mM Tris-HCl at pH 8.0 was added, and the mixture was agitated vigorously using a stirring centrifuge and centrifuged for 5 min at 15,000 × *g*, after which the supernatant was removed to wash the pulpy DBS (Step 4). Subsequently, the pulpy DBS was suspended by adding 200 μL of 50 mM Tris-HCl pH 8.0 and agitating vigorously for 3 min at maximum using a stirring centrifuge (Step 5).

**Figure 1:**
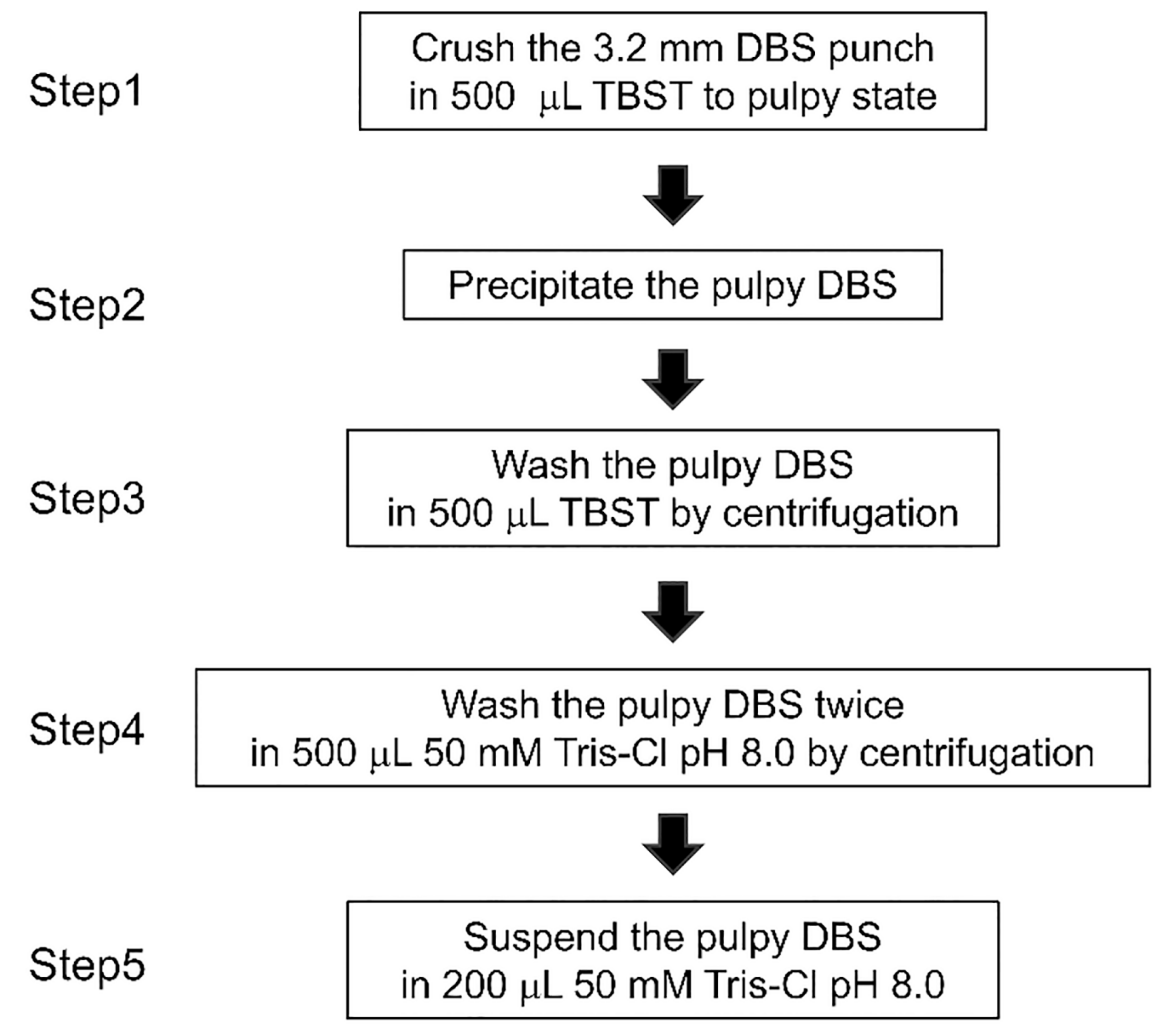
Flowchart of the preparation of proteins for nontargeted analysis of nonspecifically dried blood spot-absorbed proteins (NANDA).

### Automated Protein Preparation Using NANDA

The automated preparation of proteins for NANDA is shown in Figure 2 and Video S1. Ninety-six-well plates for Maelstrom 8-Autostage/Maelstrom 9610 instrument (TANBead) processing were prepared. Briefly, 500 μL of 1× TBST, 5 mg of freshly prepared iron powder solution (3–5 μm particle size purchased from Kojundo Chemical Lab. Co., Ltd., Japan, 5 mg/25 μL in 50% glycerol), and one 3.2 mm-diameter disk punched out from DBS was added to the first row/first 96-well plate. Next, 500 μL of 1× TBST was added to the second row/second 96-well plate, and 500 μL of 50 mM Tris-HCl at pH 8.0 was added to the third and fourth row/third and fourth 96-well plates. Finally, 200 μL of 50 mM Tris-HCl at pH 8.0 was added to the fifth row/fifth 96-well plate. Subsequently, the 96-well plate was set on the Maelstrom 8-Autostage/Maelstrom 9610, and the protein extraction procedure from DBS was initiated. A spin tip was inserted into the first row/plate, and the DBS was crushed by agitating in TBST for 30 min at 3,500 rpm with inverting every 10 s. This formed a complex of pulp-like DBS and iron powder. Next, a magnetic rod was inserted into the spin tip, and the complex was adsorbed and collected on the spin tip over 60 s before being moved to the second row/second plate containing TBST. After removing the magnet rod from the spin tip in TBST, the complex was washed by agitation for 10 min at 3,500 rpm with inverting every 10 s. Afterward, the complex was transferred to the third row/third plate containing 50 mM Tris-HCl at pH 8.0 and washed by agitating the spin tip for 10 min at 3,500 rpm with inverting every 10 s. This washing process was repeated in the fourth row/fourth plate containing 50 mM Tris-HCl pH 8.0 by agitating the spin tip for 2 min at 3,500 rpm with inverting every 10 s. Finally, the complexes were transferred to the fifth row/fifth plate containing 50 mM Tris-HCl at pH 8.0 and suspended by agitating the spin tip for 2 min at 3,500 rpm with inverting every 10 s.

**Figure 2:**
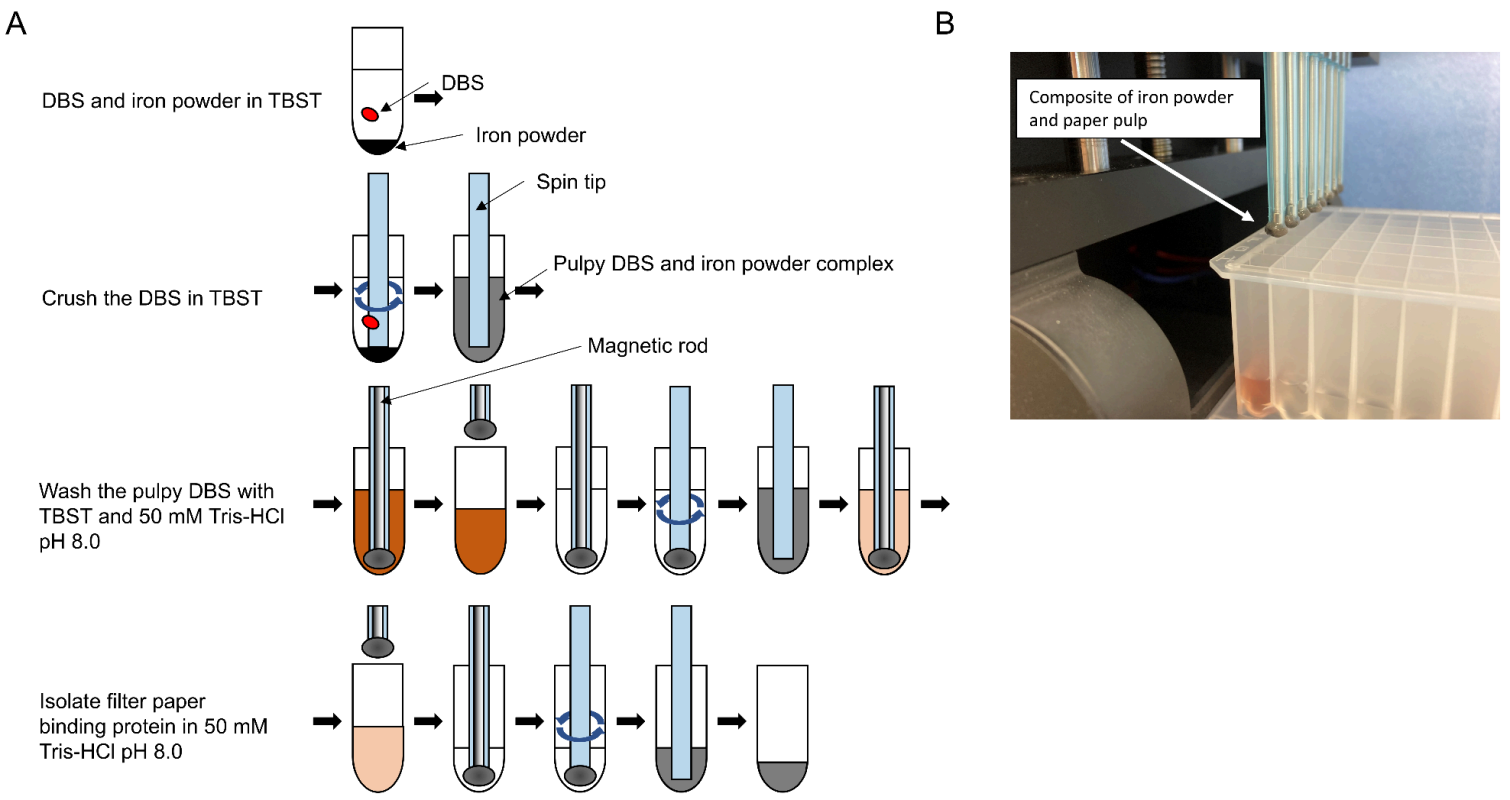
Automated preparation of proteins for nontargeted analysis of NANDA. (A) Preparation of proteins for automated (auto-NANDA) using Maelstrom 8-Autostage/Maelstrom 9610. (B) Pulpy DBS and iron powder complex isolated by a magnet.

### Protein Digestion

For protein digestion, 1 μg of trypsin/Lys-C Mix (CAT# V5072; Promega, Madison, WI, USA) was gently mixed with the sample for 14 h at 37 °C. After removing the filter paper residue from the digested sample using Maelstrom 8-Autostage/Maelstrom 9610, the isolated supernatant was acidified with 50 μL of 5% trifluoroacetic acid (TFA). Thereafter, the sample was desalted using a styrene–divinylbenzene polymer stop-and-go extraction tip (SDB-STAGE tip; GL Sciences, Tokyo, Japan), which was washed with 25 μL of 80% acetonitrile (ACN) in 0.1% TFA, followed by equilibration with 50 μL of 3% ACN in 0.1% TFA. Next, the sample was loaded onto the tip, washed with 80 μL of 3% ACN in 0.1% TFA, and eluted with 50 μL of 36% ACN in 0.1% TFA. The eluate was dried in a centrifugal evaporator (miVac Duo concentrator; Genevac, Ipswich, UK). The dried sample was then redissolved in 0.02% decyl maltose neopentyl glycol (Anatrace Products, LLC, Maumee, OH, USA). Subsequently, the redissolved sample was assayed for peptide concentration using a Lunatic instrument (Unchained Laboratories, Pleasanton, CA, USA) and transferred to an LC vial (Thermo Fisher Scientific, Waltham, MA, USA). Additionally, we omitted the reduction and alkylation steps to streamline the process. For the DBS protein, we could not examine alkylation before trypsin digestion because of the effects of filter paper residue and iron powder. Instead, we first examined the effects of no alkylation, alkylation before trypsin digestion, and alkylation after trypsin digestion on HEK 293 protein. The results demonstrated that the highest number of proteins were identified when alkylation was performed after protein digestion (Figure S1). Subsequently, a comparison of the number of identified DBS proteins under nonalkylated conditions and postdigestion alkylation revealed that, although the number of identified peptides slightly decreased without these steps, the number of identified proteins did not decrease (Figure S2A,B and Table S1). Furthermore, more than 98% of proteins and 80% of peptides detected under alkylation conditions were also detected in the absence of alkylation (Figure S2C,D). Considering the need to process many samples, skipping the reduction and alkylation steps was beneficial.

### LC–MS/MS with DIA

For DBS protein analysis, the redissolved peptides were injected directly into a 75 μm × 25 cm nanoLC column (Aurora C18, particle size 1.6 μm, 120 Å; IonOpticks, Fitzroy, VIC, Australia) at 60 °C and separated using a 0 min gradient (A = 0.1% formic acid in water, B = 0.1% formic acid in 80% ACN with the following settings: 0 min at 4% B, 78 min at 32% B, 84 min at 75% B, and 90 min at 75% B, all at a flow rate of 200 nL/min. This separation was performed using an UltiMate 3000 RSLCnano LC system (Thermo Fisher Scientific). The eluting peptides from the column were then analyzed on an Orbitrap Exploris 480 MS (Thermo Fisher Scientific) equipped with an InSpIon system (AMR, Tokyo, Japan).^9^ For DIA, the precursor range was defined based on previously established parameters.^10^ MS1 spectra were collected at nine different ranges corresponding to the DIA method (395–605, 395–705, 395–805, 495–705, 495–805, 495–905, 595–805, 595–905, and 695–905 *m*/*z*) at a resolution of 15,000, with an automatic gain control target of 3 × 10^6^ and a maximum injection time of “Auto”. MS2 spectra were collected in the range of 200–1,800 *m*/*z* at a resolution of 30,000, with an automatic gain control target of 3 × 10^6^, a maximum injection time set to “Auto,” and stepped normalized collision energies of 22, 26, and 30%. For the DIA window patterns (400–600, 400–700, 400–800, 500–700, 500–800, 500–900, 600–800, 600–900, and 700–900 *m*/*z*), an optimized window arrangement was employed using Xcalibur 4.3 (Thermo Fisher Scientific). The isolation width for MS2 was set to 4, 6, and 8 Th at 200, 300, and 400 *m*/*z* with the DIA mass range, respectively.

In the analysis using the Orbitrap Astral MS (Thermo Fisher Scientific), the redissolved peptides were injected directly onto a 75 μm × 30 cm nanoLC column (ReproSil-Pur C18, particle size 1.5 μm, 100 Å; CoAnn Technologies, Richland, WA, USA) at 60 °C, and separated using a 84.5 min gradient (15 samples/day, SPD) comprising 1% B at a flow rate of 600 nL/min in 0–0.5 min, 1–8% B at a flow rate of 600–200 nL/min in 0.5–4.5 min, 8–23% B at a flow rate of 200 nL/min in 4.5–60 min, 23–40% B at a flow rate of 200 nL/min in 60–78.5 min, 40–98% B at a flow rate of 200 nL/min in 78.5–79.5 min, 98% B at a flow rate of 200 nL/min in 79.5–80.5 min, 98% B at a flow rate of 200–600 nL/min in 80.5–81.5 min, 98% B at a flow rate of 600–750 nL/min in 81.5–82.5 min, and 98% B at a flow rate of 750 nL/min in 82.5–84.5 min; a 23 min gradient (45 SPD) comprising 1% B at a flow rate of 700 nL/min in 0–0.5 min, 1–10% B at a flow rate of 700–400 nL/min in 0.5–3.5 min, 10–28% B at a flow rate of 400 nL/min in 3.5–15.5 min, 28–42% B at a flow rate of 400 nL/min in 15.5–19.5 min, 42–98% B at a flow rate of 400 nL/min in 19.5–20.5 min, 98% B at a flow rate of 400 nL/min in 20.5–21.5 min, 98% B at a flow rate of 400–800 nL/min in 21.5–22.5 min, and 98% B at a flow rate of 800 nL/min in 22.5–23 min. Subsequently, the eluting peptides from the column were analyzed on the Orbitrap Astral MS (Thermo Fisher Scientific) equipped with an InSpIon system. MS1 spectra were collected in the range of *m*/*z* 380–980 at a 240,000 resolution using the Orbitrap to set an automatic gain control target of 500% and a maximum injection time of 5 ms. MS2 spectra were collected at *m*/*z* 250–2,000 and 200–2,000 using an the Orbitrap Astral analyzer to set an automatic gain control target of 600 and 500%, maximum injection time of 4 and 3 ms for 15 and 45 SPD, respectively, and a normalized collision energy of 25%. The isolation width for MS2 was set to 1.6 and 3 Th for 15 and 45 SPD, respectively.

For HEK 293 protein analysis, the redissolved peptides were injected directly into a 75 μm × 30 cm nanoLC column (ReproSil-Pur C18, particle size 1.7 μm, 100 Å; CoAnn Technologies, Richland, WA, USA) at 60 °C, and separated using a 110 min gradient with the following settings: 2% B at flow rate of 500 nL/min in 0–16 min, 2–6% B at flow rate of 150 nL/min in 16–20 min, 6–34% B at flow rate of 150 nL/min in 20–96 min, 34–70% B at flow rate of 150 nL/min in 96–103 min, 70% B at flow rate of 150 nL/min in 103–110 min. This separation was performed using an UltiMate 3000 RSLCnano LC system (Thermo Fisher Scientific). The eluting peptides from the column were then analyzed on the Orbitrap Exploris 480 MS equipped with an InSpIon system (Thermo Fisher Scientific). For DIA method, the method duration was set to 90 min. MS1 spectra were collected in the range of *m*/*z* 495–705 at a resolution of 15,000, with an automatic gain control target of 300% and a maximum injection time of “Auto.” MS2 spectra were collected in the range of 200–1,800 *m*/*z* at a resolution of 60,000, with an automatic gain control target of 3000%, a maximum injection time set to “Auto,” and stepped normalized collision energies of 22, 26, and 30%. For the DIA window patterns (500–700 *m*/*z*), an optimized window arrangement was employed using Xcalibur 4.3 (Thermo Fisher Scientific). The isolation width for MS2 was set to 4 Th.

### Data Analysis

For DBS protein analysis, The DIA-MS data were queried against the *in silico* human spectral library using DIA-NN v.1.9.1 (https://github.com/vdemichev/DiaNN).^11^ Initially, the spectral library was generated from the human protein sequence UniProt database (proteome ID UP000005640, 20,598 entries, downloaded on April 1, 2024) using DIA-NN. The parameters for generating the spectral library were as follows: digestion enzyme, trypsin; missed cleavages, 1; peptide length range, 7–45; precursor charge range, 2–4; and fragment ion *m*/*z* range, 200–1,800. The precursor mass range was varied according to the DIA method (400–600, 400–700, 400–800, 500–700, 500–800, 500–900, 600–800, 600–900, and 700–900 *m*/*z*). Additionally, “FASTA digest for library-free search/library generation,” “deep learning-based spectra, RTs and IM prediction,” and “n-term M excision” were enabled. For the DIA-NN search, the following parameters were applied: mass accuracy of 10 ppm, MS1 accuracy of 10 ppm, protein inference based on genes, utilization of neural network classifiers in single-pass mode, quantification strategy using QuantUMS (high precision), and cross-run normalization set to “RT-dependent.” Furthermore, “Unrelated runs,” “Peptideforms,” “Heuristic protein inference,” and “No shared spectra” were enabled, whereas match-between-run was deactivated. The threshold for protein identification was set to 1% or less for both precursor and protein false discovery rates. The protein quantification values were aggregated over the quantification values of unique peptides as calculated by DIA-NN. A similar pipeline was used for the HEK 293 protein analysis. However, “C Carbamidomethylation” was enabled as a fixed modification only for the analysis of reduced and alkylated samples.

Log2 transformation of protein intensity was performed, and a filtering step was conducted to ensure that, for each protein, at least one group contained a minimum of 70% valid values. Missing values were imputed using random numbers drawn from a normal distribution (width, 0.3; downshift, 1.8) using Perseus v1.6.15.0,^12^ and Pearson’s correlation analysis was performed. Gene ontology enrichment analysis for matching to the Online Mendelian Inheritance in Man (OMIM) database were conducted using DAVID (https://david.ncifcrf.gov/tools.jsp). Other graphs were generated using GraphPad Prism 9 (GraphPad Software, San Diego, CA, USA) or Excel (Microsoft Corporation, Redmond, WA, USA).

## Results and Discussion

### Proof-of-Concept Experiment of the Nontargeted Proteome Analysis of Filter Paper-Retained Proteins

In our previous study, we removed abundant soluble serum/plasma proteins from DBS extracts using salt-induced precipitation.^13^ However, we considered further improvements in protein coverage depth and throughput mandatory for implementing nontargeted proteome analysis in real-world NBS. In the trial-and-error processes to this end, we noticed that considerable amounts of proteins remained on DBS, even after extensive washing with buffer solution. This finding suggests that we could use the filter paper used for DBS as a solid support that absorbs proteins (probably with low solubility) nonspecifically. If this is the case, simple washing of DBS with an appropriate buffer might enable us to remove a large amount of soluble serum/plasma proteins without losing low-solubility proteins on the filter paper. To pursue this possibility, we conducted a proof-of-concept experiment as described below.

In this context, we devised a NANDA, as shown in Figure 1. In NANDA, DBS was first suspended in TBST until it became a suspended paper pulp and was then centrifuged to precipitate the paper pulp. Next, the precipitate was washed once with TBST and twice with 50 mM Tris-HCl at pH 8.0 to separate the filter paper debris from which soluble proteins, such as albumin, had been removed. As a considerable amount of proteins were kept bound to the filter paper pulp, even after extensive washing with TBST, we directly added protease to this pulp suspended in 50 mM Tris-HCl at pH 8.0 for digestion and finally yielded approximately 2–5 μg of digested peptides in the suspension. The DIA–LC–MS/MS data of the peptide mixtures thus obtained indicated that NANDA allowed us to identify approximately 1.8-fold (3,541/1,925) more proteins (number of protein groups stably detected in four replicates by each method) than the sodium carbonate precipitation (SCP) method reported in 2020 ^13^ (Figure 3A). Over 93% (1,809 out of 1,925) of the proteins identified by the SCP method were also detected using NANDA (Figure 3B).

**Figure 3:**
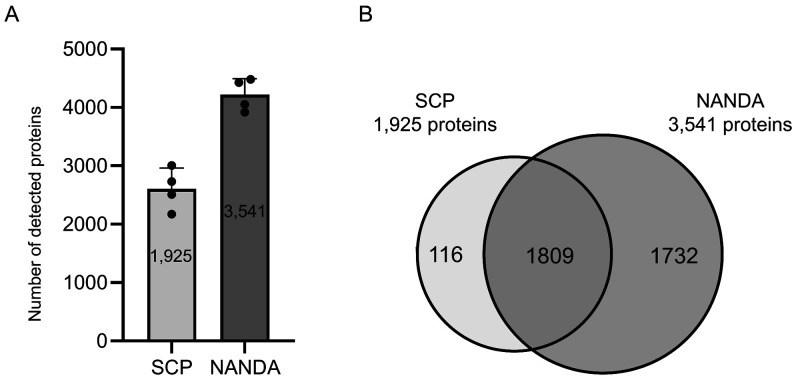
Number of proteins detected using sodium carbonate precipitation (SCP) and NANDA. (A) Number of proteins detected using SCP and NANDA (*n* = 4). The number in the bar graph shows the number of proteins commonly detected in four replicates. (B) Comparison of proteins detection by the two preparations. The number in the Venn diagram indicates the number of proteins commonly detected in the four replicates.

The data described above demonstrated that NANDA was simpler and less labor-intensive, but provided deeper protein coverage than the SCP method. In this regard, it should be noted that the analysis of nonspecifically bound proteins on DBS is not a new concept in literature: Molloy et al. reported that a simple DBS washing step using a sophisticated volumetric absorptive microsampling device (VAMS) made it possible to robustly quantify up to 1,600 proteins from single-shot shotgun LC–MS/MS analysis.^14^ The methodological differences between NANDA and their report included differences in solid support (conventional filter paper vs VAMS support) and washing conditions. Regarding the solid support, we confirmed that NANDA provided the same results using conventional filter papers used for NBS supplied by three different suppliers (Figure S3), although we did not check the solid support we used in VAMS. Regarding the washing conditions, DBS was subjected to more extensive washing in NANDA as a suspended paper pulp than in the method by Molloy et al.^14^ We assume that the complete removal of contaminating abundant plasma proteins must contribute to deepening the identified protein coverage compared with that in the method by Molloy et al.^14^ Furthermore, Molloy et al. utilized the Q Exactive HF-X MS (Thermo Fisher Scientific) operating in DDA mode, whereas our approach employs the latest high-sensitivity Orbitrap Exploris 480 MS and Orbitrap Astral MS in DIA mode. The DIA mode surpasses DDA due to its superior sensitivity, specificity, and reproducibility for protein identification.^15–17^ Unlike DDA, DIA comprehensively analyzes all MS/MS fragment ions within a predefined mass range, ensuring the detection of low-abundance peptides and more precise quantitative analysis. Together with optimizing the precursor MS range and isolation window width (Figure S4), the number of identified protein groups exceeded 5,000 using NANDA coupled with the DIA–LC–MS/MS system.

After confirming the concept of NANDA, we next sought to optimize and develop a robotic system for NANDA to improve overall throughput. The characteristics of the protein groups detected using NANDA are described in detail in the subsequent sections.

### Development and Validation of Automated NANDA Platform

#### Centrifugation-Free Washing of Filter Paper Pulp Using Low-Cost Iron Powders

To improve NANDA throughput, we attempted to develop a centrifugation-free system. As the inclusion of high-speed centrifugation in NANDA made it difficult to build a high-throughput sample-processing platform, we aimed to develop an alternative to washing dispersed paper pulp without centrifugation steps. After much trial and error, we finally arrived at a method that enabled the manipulation of suspended paper pulp using magnetism instead of centrifugation. In this system, we used iron powder as a cost-effective magnetic carrier to form the suspended paper pulp into a bundle. A video showing this process is available (Video S1). Next, we implemented the iron powder-assisted NANDA (auto-NANDA) on a robotic processing device for magnetic beads (Maelstrom 8-Autostage or Maelstrom 9610; TANBead, Taoyuan City, Taiwan). Figure 2A shows the auto-NANDA scheme. By adding iron powder during DBS suspension in the initial TBST, a mixture of iron powder and dispersed paper pulp was produced automatically by the rotational motion of the spin tip. By inserting a magnetic rod into the spin tip, this complex was magnetically bound to the spin tip, allowing for easy and almost complete isolation. The iron powder and paper pulp composite was bound more compactly to the spin tip than to the centrifugation-assisted collection of suspended paper pulp, resulting in less carryover of washing solution retained by the paper pulp. This certainly improved the washing efficiency (Figure 2B). The composite was then transferred to a new well and washed once with TBST and twice with 50 mM Tris-HCl at pH 8.0 using the spin tip’s rotation and magnetic manipulation. Finally, the composites were robotically transferred to a trypsin-digestible solution. Once the reagents were set in a 96-well plate, the entire process took approximately 1 h, with Maelstrom 8-Autostage processing for 8 samples and Maelstrom 9610 processing for 96 samples simultaneously.

#### Auto-NANDA Cost Issue

The washed composite was directly subjected to digestion with peptidase (in our case, trypsin). After digestion, the digested peptides released from the composite were separated and retrieved from the iron powder and filter paper debris using the Maelstrom devices. The samples were then desalted simultaneously using a stage-tip adapter. As peptidase accounts for approximately 40% of the overall running cost of auto-NANDA, we manually added peptidase using an Eppendorf electronic continuous pipet to decrease the running cost. Although peptidase can be added robotically, it inevitably results in wastage in the dead volume of the robotic dispensing system. Therefore, by adding peptidase manually, the cost per sample when simultaneously processing 96 samples was approximately $3, making it acceptable for application in NBS.

#### Comparison and Validation of the Performances of auto-NANDA

We subsequently compared the performance of SCP, manual NANDA, and auto-NANDA. Manual NANDA identified 3,541 protein groups stably detected in four replications, while auto-NANDA identified 4,692 protein groups (Figure 4A). To investigate why auto-NANDA identified more proteins, we compared the total intensities of hemoglobin subunits α (HBA2) and β (HBB), the most abundant proteins in erythrocytes (Figure 4B). These proteins can interfere with the detection of low-abundance proteins, as major plasma proteins, such as albumin, were extensively removed by both methods. In auto-NANDA, the ion intensity of hemoglobin decreased to approximately 60% compared with that in the manual method. The observed signal reduction of hemoglobin likely contributed to the increased detection of low-abundance proteins. In auto-NANDA, the direct collision of the filter paper with the spin tip, along with the periodic reversal of the spin tip’s direction (unique features of Maelstrom 8-Autostage and Maelstrom 9610), likely enhanced washing efficiency.

**Figure 4:**
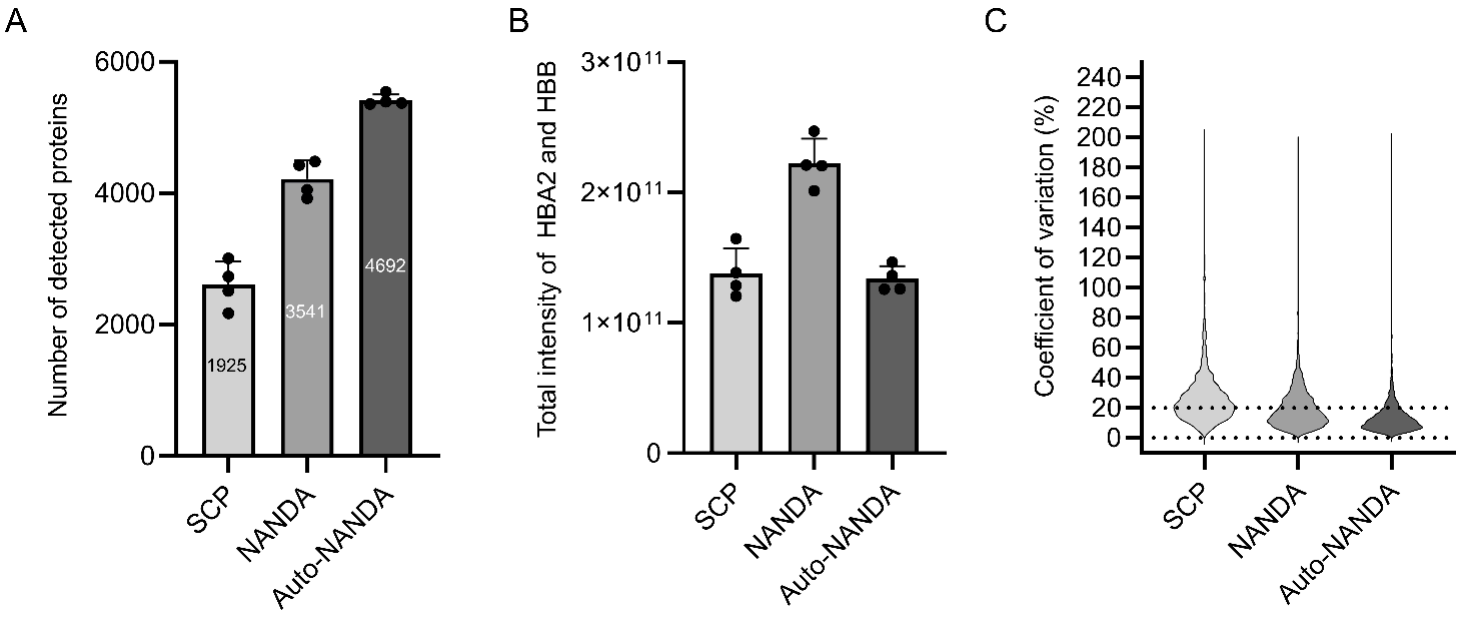
Comparison of proteins detected by manual and automated nonspecifically dried blood spot-absorbed proteins. (A) Number of proteins detected by SCP, NANDA, and auto-NANDA (*n* = 4). The numbers in the bar graph represent the number of proteins commonly detected in four replicates. (B) Total ion intensity of hemoglobin subunits α (HBA2) and β (HBB) detected by SCP, NANDA, and auto-NANDA (*n* = 4). (C) Distribution of the coefficient of variation (CV) of ion intensities of proteins commonly detected in four replicates.

The automated method detected a similar number of proteins from DBS using different filter papers (Whatman 903 Protein Saver Cards; Whatman, Springfield Mill, UK, and PerkinElmer 226 Spot Saver Card; PerkinElmer. Waltham, MA, USA) as it did with the ADVANTEC PKU-S (ADVANTEC, Tokyo, Japan) (Figure S3). Furthermore, to evaluate the reproducibility of protein detection and quantification between manual and automated methods, the CV values for the ion intensities of individual proteins were analyzed (Figure 4C). The numbers of proteins with CV values <20% were 2,250 and 3,998 for the manual and automated methods, respectively. Automation facilitated highly reproducible sample preparation, significantly increasing the number of consistently detectable proteins. The lower reproducibility of auto-NANDA than that of conventional proteome analysis using bulk cultured cells^10^ may be attributed to sampling variations depending on the DBS punching location. The results indicated that auto-NANDA considerably improved throughput, proteome depth, and sample preparation robustness, making it suitable for NBS. These benefits ensure consistent and stable results, regardless of when, where, or by whom DBS analyses are conducted, which is crucial for clinical testing applications.

### Feasibility of NBS using auto-NANDA

#### Coverage of Disease-Relevant Proteins by auto-NANDA

To assess the applicability of NANDA for NBS, we examined how SCP, manual NANDA, and auto-NANDA detect several disease-relevant proteins in the OMIM database (Figure 5). As shown in Figure 5, SCP, manual NANDA, and auto-NANDA detected 975, 1,540, and 1,864 OMIM proteins, respectively, indicating that auto-NANDA enabled us to detect 1.9-fold more OMIM proteins than the SCP method. As we intend to use this method for NBS in the future, we next compared the detection performance of gene products explicitly listed in the recommended uniform screening panel by the Health Resources & Services Administration (https://www.hrsa.gov/advisory-committees/heritable-disorders/rusp). The data showed that auto-NANDA increased the number of detected gene products to 36, while the SCP method could only detect 17 out of 85 listed gene products (counted only gene products without missing values in protein quantification among *n* = 4, Table S2). These results suggest that auto-NANDA considerably improved the applicability of the nontargeted proteome approach for NBS.

**Figure 5:**
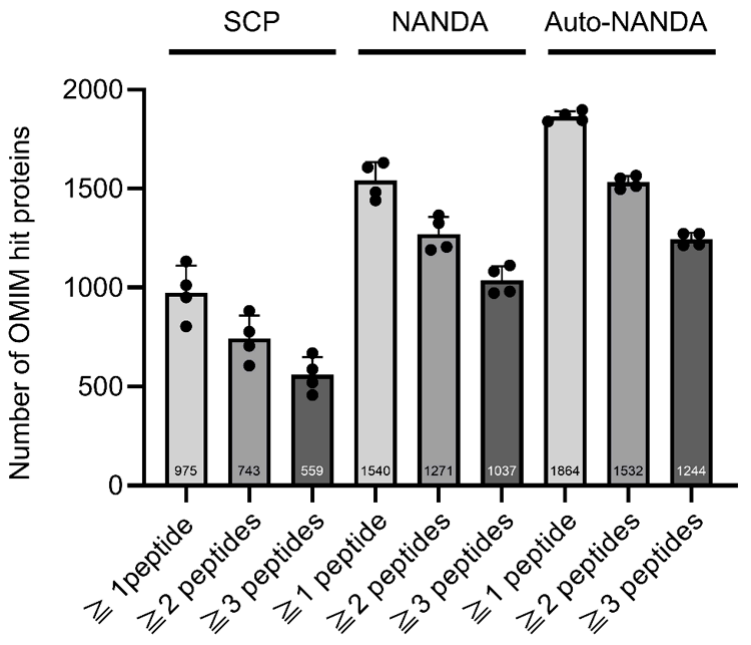
Comparison of the numbers of the Online Mendelian Inheritance in Man hit proteins among different methods. Methods compared are SCP,NANDA, auto-NANDA with peptide number filters from 1 to 3 (*n* = 4). The numbers displayed in the bar graph indicate the average number of proteins detected in each of the four replicate experiments.

In previous discussions of proteins detectable via DBS proteome analysis, we included those identified only by a single peptide. However, for practical NBS applications, single peptide detections are insufficiently robust if they are polymorphic with missense mutations. Therefore, we investigated how the number of proteins detected using two or more peptides varied across SCP, NANDA, and auto-NANDA (Figure 5). The results showed that for the SCP method, 76% (743/975) and 57% (559/975) of proteins were detected using ≥ 2 and ≥ 3 peptides, respectively. In manual NANDA, 83% (1,271/1,540) were detected using ≥ 2 peptides, and 67% (1037/1540) using ≥ 3 peptides. In Auto-NANDA, 82% (1,532/1,864) were detected using ≥ 2 peptides and 67% (1244/1864) using ≥ 3 peptides. Manual and auto-NANDA resulted in a smaller decrease in the number of detected proteins with an increasing number of detected peptides than the SCP method. Notably, the ATP7B protein, a causative gene product of Wilson disease, was detected only after antibody-assisted enrichment^18^ and only with two peptides using the SCP method. However, it was stably detected with ≥ 3 peptides using auto-NANDA. In this regard, auto-NANDA detected approximately twice as many OMIM-registered proteins as the SCP method, implying its higher potential for deep screening for various genetic disorders (Figure 5). The proteins detected by SCP, manual NANDA, and auto-NANDA are listed in Table S2.

#### Data Reproducibility

We confirmed the daily and interdevice reproducibility of auto-NANDA, because reproducibility of the measurement platform is critical for screening (Figure 6). We independently prepared samples from 12 DBS disks using auto-NANDA thrice at different time points (days 1, 32, and 60). These samples were then analyzed using DIA-MS on two different LC–MS instruments with the same specifications and settings. The Pearson’s correlation coefficient values for the 72 data sets had a median of 0.99 and minimum of 0.98, demonstrating high reproducibility of overall NANDA (Figure 6). Even if protein extraction was conducted on a different day, the Pearson’s correlation coefficient value was consistently ≥0.98. This demonstrated the high reproducibility of protein extraction across different days, considering the variability in DBS itself, subsequent protein extraction, protein digestion, desalting, and LC–MS/MS analysis. Additionally, instruments of the same model were confirmed to produce highly correlated data. We thus considered that the overall performance of auto-NANDA coupled with DIA–LC–MS/MS is suitable for large-scale NBS and cohort studies.

**Figure 6:**
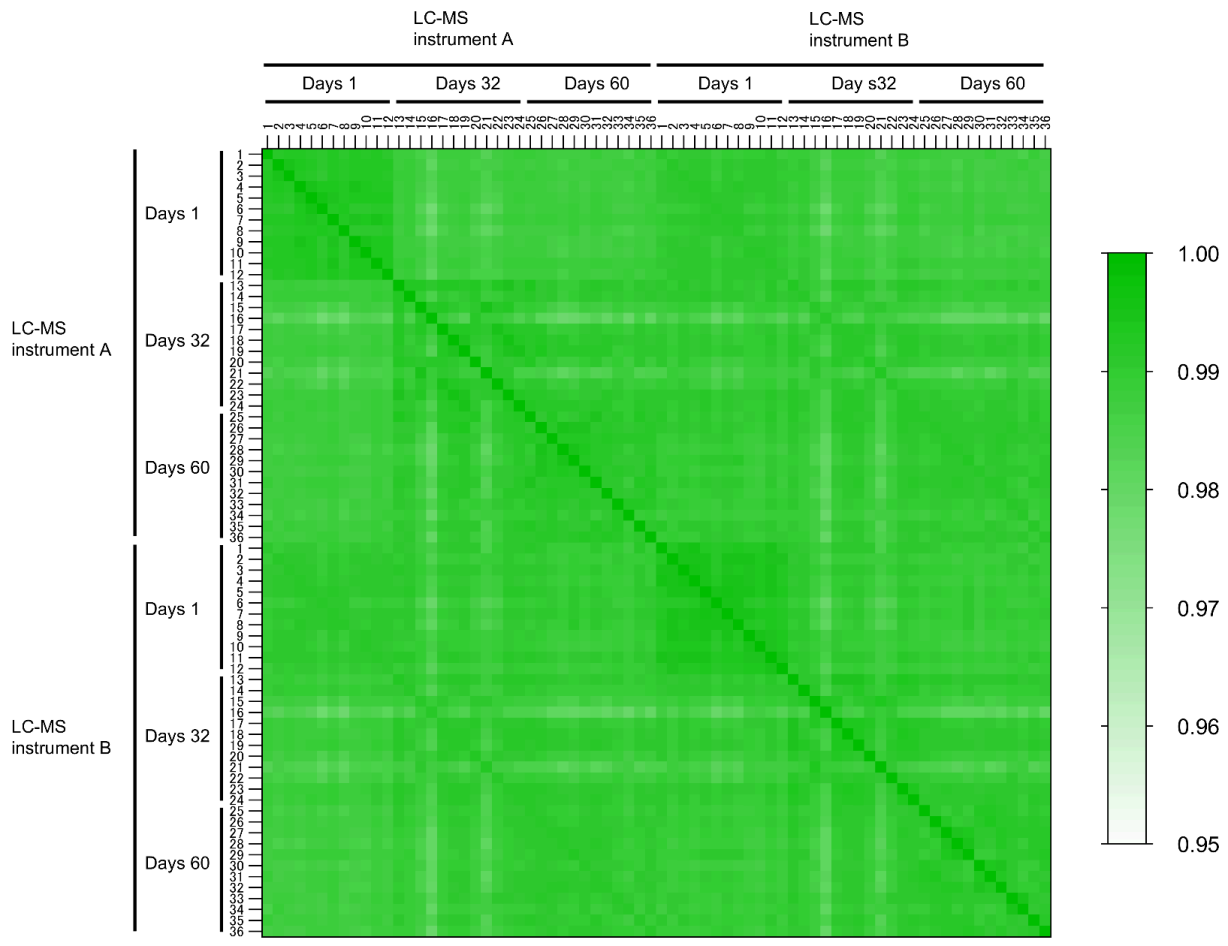
Reproducibility between LC–MS instruments and days in automated NANDA. Pearson’s correlation analysis of protein intensities of samples 1–12, 13–24, and 25–36 prepared using auto-NANDA. The methods on days 1, 32, and 60 were tested using LC–MS instruments A and B with the same performance.

Furthermore, considering the practical application to newborn screening, a total of 96 samples were independently prepared in three batches of 24 DBS disks each (1–32, 33–64, and 65–96) using auto-NANDA and analyzed continuously via LC–MS with the Orbitrap Astral MS 45 SPD. The resulting data exhibited a high correlation, with a median Pearson’s correlation coefficient value of 0.97 (Figure S5). However, further evaluation is necessary to determine whether this level of reproducibility is sufficient for actual disease screening.

#### Turnaround Time of the DIA–LC–MS/MS Step

We already demonstrated the feasibility of NBS using DBS proteome analysis with the SCP method for genetic disorders.^8^ Examining proteins, which are the end products of genes, can complement genetic testing, and the demand for such proteomics approaches is growing. Although the SCP method had throughput- and reproducibility-related issues because of the lack of automation, auto-NANDA solved most of these issues.

One of the remaining issues is the turn-around time of the DIA–LC–MS/MS step. Given that nontargeted proteome analysis is conducted individually, the time required for a single DIA–LC–MS/MS analysis greatly impacts the throughput of NBS. Therefore, we explored the point of compromise between sensitivity and analysis time using the latest Orbitrap Astral MS (Thermo Fisher Scientific; Figure 7). Using the Orbitrap Exploris 480 MS (Thermo Fisher Scientific), the number of detected proteins was 5,415 in a 90 min gradient of analysis (12 SPD including overhead time), while the Orbitrap Astral MS detected 6,359 proteins in a shorter time of an 84 min gradient, i.e., 15 SPD including overhead time. Additionally, the reduction in the average number of detected proteins when increasing the threshold number of detected peptides to two and three was less pronounced with the Orbitrap Astral MS (88% [5,776/6,539] and 76% [4,990/6,539], respectively) than that with the Orbitrap Exploris 480 MS (78% [4,224/5,415] and 60% [3,265/5,415], respectively). Furthermore, the analysis time was reduced to just a 23 min gradient (45 SPD including overhead time), and despite considering only proteins identified with three or more peptides while accounting for polymorphic variations with missense mutations, the total number of such proteins reached 3,531 surpassing the 3,265 proteins detected in the 90 min analysis using the Orbitrap Exploris 480 MS (Table S3). Based on this, considering the detection of polymorphic variations with missense mutations, the 45 SPD method using the Orbitrap Astral MS offers advantages over the 12 SPD method using the Orbitrap Exploris 480 MS, and by utilizing state-of-the-art MS, it has demonstrated the potential for applicability to newborn screening even with a shortened MS analysis time.

**Figure 7:**
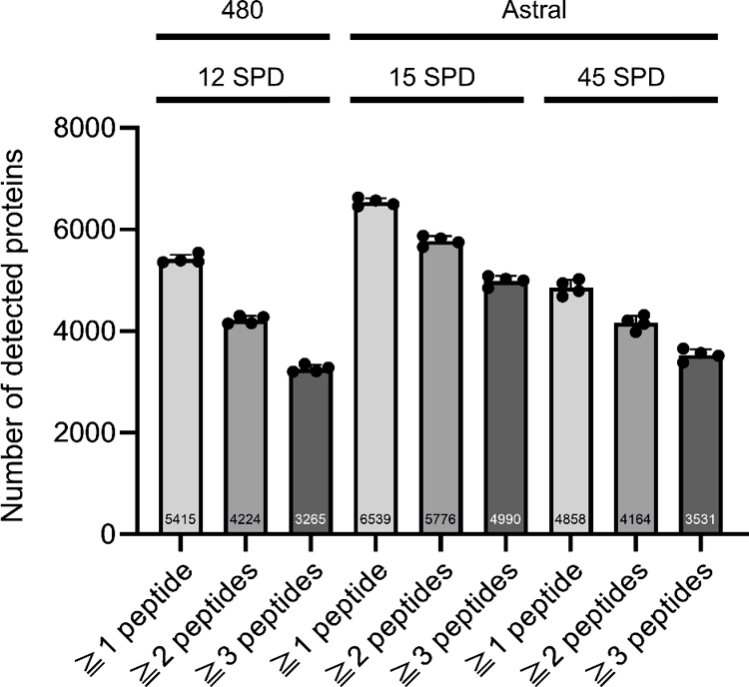
Numbers of proteins detected using different methods. Methods compared are auto-NANDA with an Orbitrap Exploris 480 MS (12 SPD) and Orbitrap Astral MS (45 and 15 SPD), categorized by the number of detected peptides per protein (*n* = 4). The numbers displayed in the bar graph indicate the average number of proteins detected in each of the four replicate experiments.

We developed a 45 SPD analytical method using the Orbitrap Astral MS to process 10,000 samples annually for newborn screening. With the 45 SPD method, a simple calculation shows that 16,425 samples can be analyzed annually. However, in practical newborn screening, sample carryover presents a critical issue, necessitating blank measurements between analyses, even if they are brief. When incorporating short blank measurements, the total measurement time per sample increases to approximately 40 min, thereby reducing the estimated throughput to 13,140 samples per year. Furthermore, given LC–MS instrument downtime due to routine calibration and maintenance, the annual utilization rate of the LC–MS instrument is approximately 80%, resulting in a final capacity of 10,512 samples per year. Based on these estimates, it is predicted that this system can perform over 10,000 newborn screening tests annually.

#### Current Limitations of the Nontargeted Proteome Approach by the DIA–LC–MS/MS

We recently reported the feasibility of NBS using quantitative DBS proteome analysis with the SCP method for genetic disorders.^8^ Although proteome analysis can potentially identify polymorphisms in protein sequences if the protein of interest is sufficiently abundant,^19^ the concept validated by this study focused solely on quantitative protein profiling. This approach is well-suited for detecting genetic disease caused by mutations resulting in a complete protein-null molecular phenotype. That is, diseases with dominant inheritance are currently difficult to detect using this approach. Thus, diseases screened using the nontargeted proteome approach should be carefully considered in terms of the mode of inheritance and the abundance of proteins detected. However, despite this limitation, our method provides a fundamental basis for various applications of DBS proteome analysis, including NBS. This is due to its deep protein coverage, which allows it to function as a “one-size-fits-all” platform; reasonable running costs; and fewer ethical concerns than genome sequencing, as long as only quantitative aspects of proteome profiles are analyzed. However, to apply this method in NBS, future studies must thoroughly examine its clinical validity and utility, along with validating its specificity in NBS.

## Conclusions

The nontargeted proteome approach has been demonstrated to possibly contribute to screening patients suffering from inborn errors of immunity with protein-null molecular phenotypes at the neonatal stage.^8^ Building on this study, we developed a robust automated method, auto-NANDA, for preprocessing proteins from DBS to enable NBS for genetic disorders using DBS protein analysis. Using auto-NANDA, the number of detected proteins from DBA increased to >5,000, while our previous SCP method enabled the detection of 1,925 proteins. We demonstrated that auto-NANDA coupled with a sophisticated DIA–LC–MS/MS system is cost-effective, highly sensitive, robust, and high-throughput, with minimal peptide detection sensitivity loss. Despite ongoing economic, ethical, and social challenges in implementing proteome-based NBS, auto-NANDA represents a major methodological breakthrough toward realizing this goal.

## Supplementary Material











## Data Availability

The LC–MS/MS data supporting the conclusions of this article are available from the ProteomeXchange Consortium in the jPOST partner repository under the accession code PXD064701.

## Notes

Identified DBS proteins with and without alkylation (XLSX)
DBS protein groups identified using SCP, NANDA, and auto-NANDA (XLSX)
DBS protein groups identified using 480-12 SPD, Astral-15 SPD and -45 SPD (XLSX)
automated preparation of proteins for NANDA­(MP4)
Number of HEK 293 proteins detected without or with reduction/alkylation, numbers of proteins and peptides detected using auto-NANDA with or without alkylation, showing comparison of numbers of proteins detected from auto-NANDA using filter paper from three manufacturers (*n* = 4), DIA-MS *m*/*z* range optimization for proteome analysis of auto-NANDA, and reproducibility of data for 96 NANDA derived from three plates (PDF)
