# Supplementary material for: Toward the Development of a Novel Newborn Screening Modality: In-Depth Nontargeted Proteome Analysis of Dried Blood Spots with a Robotic Pipeline Using Low-Cost Iron Powders
Source: Anal Chem. 2025 Aug 12;97(33):17992–8000. doi: 10.1021/acs.analchem.5c01720 (PMC12392252; doi:10.1021/acs.analchem.5c01720)
Supplement: Supplementary file 5 [file ac5c01720_si_005.pdf]

Toward the development of a novel newborn screening modality: In-depth non-targeted proteome analysis of dried blood spots with a robotic pipeline using low-cost iron powders.

Daisuke Nakajima, Masaki Ishikawa, Ryo Konno, Yusei Okuda, Hideo Sasai, Osamu Ohara, Yusuke Kawashima
Department of Applied Genomics, Kazusa DNA Research Institute

Figure S1:
Number of HEK 293 proteins detected without or with reduction/alkylation. 1

Figure S2:
Numbers of proteins and peptides detected using auto-NANDA with or without alkylation. 2

Figure S3:
Comparison of numbers of proteins detected from auto-NANDA using filter paper from three manufacturers (*n* = *4*). 3

Figure S4:
DIA-MS m/z range optimization for proteome analysis of auto-NANDA. 4

Figure S5:
Reproducibility of data for 96 NANDA derived from three plates. 5

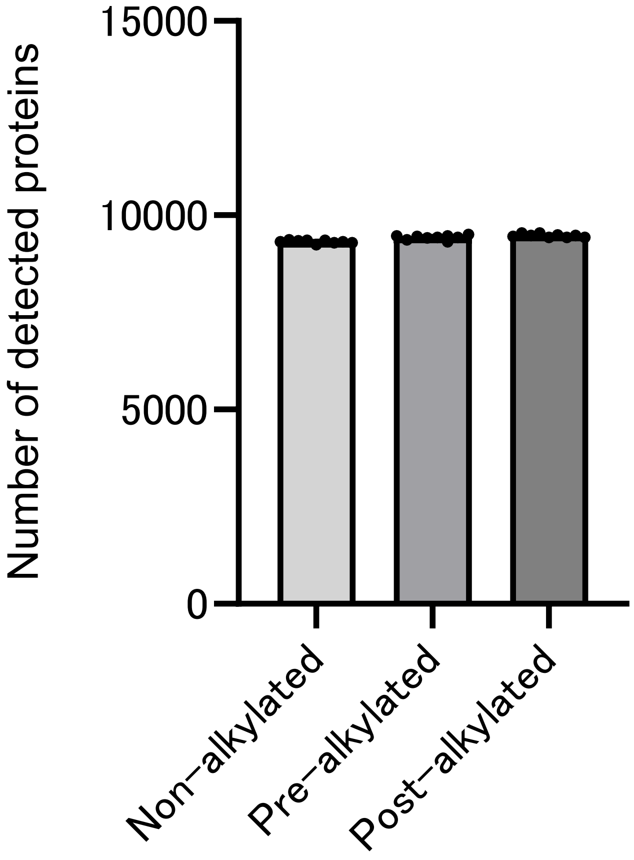

Figure S1. Number of HEK 293 proteins detected without or with reduction/alkylation

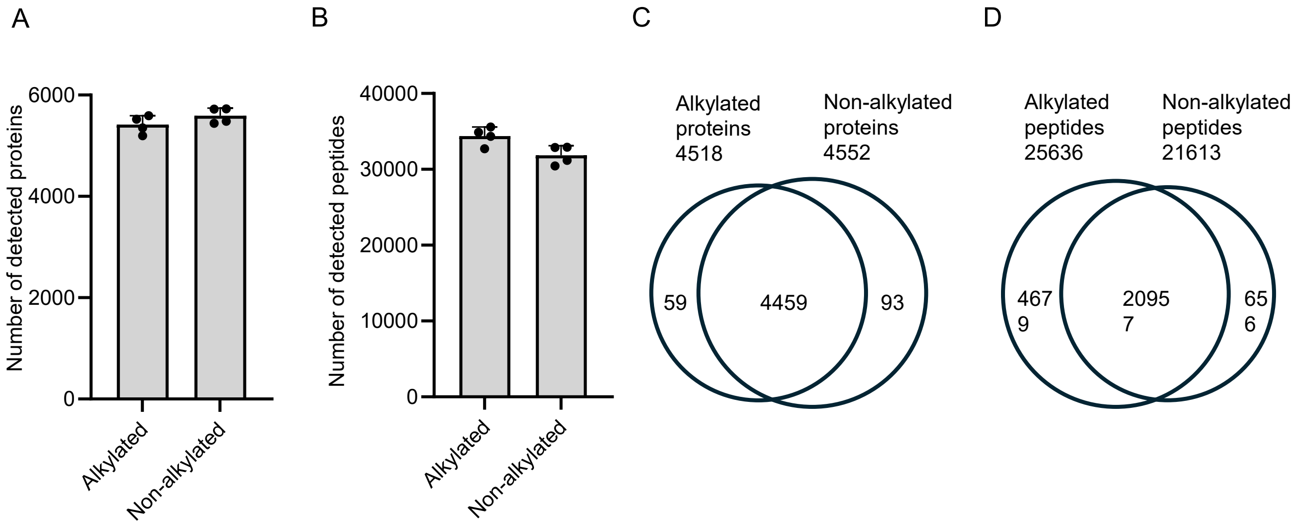

Figure S2. Numbers of proteins and peptides detected using auto-NANDA with or without alkylation. (A) Comparison of number of detected proteins. (B) Comparison of number of detected peptides. (C) Venn diagram of detected proteins commonly detected in the for replicates. (D) Venn diagram of detected peptides commonly detected in the for replicates.

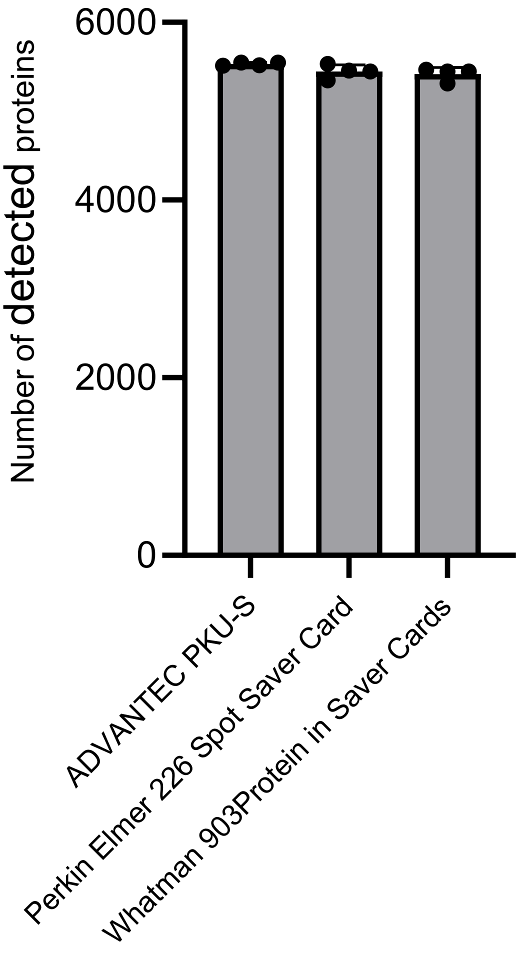

Figure S3. Comparison of numbers of proteins detected from auto-NANDA using filter paper from three manufacturers (*n* = *4*).

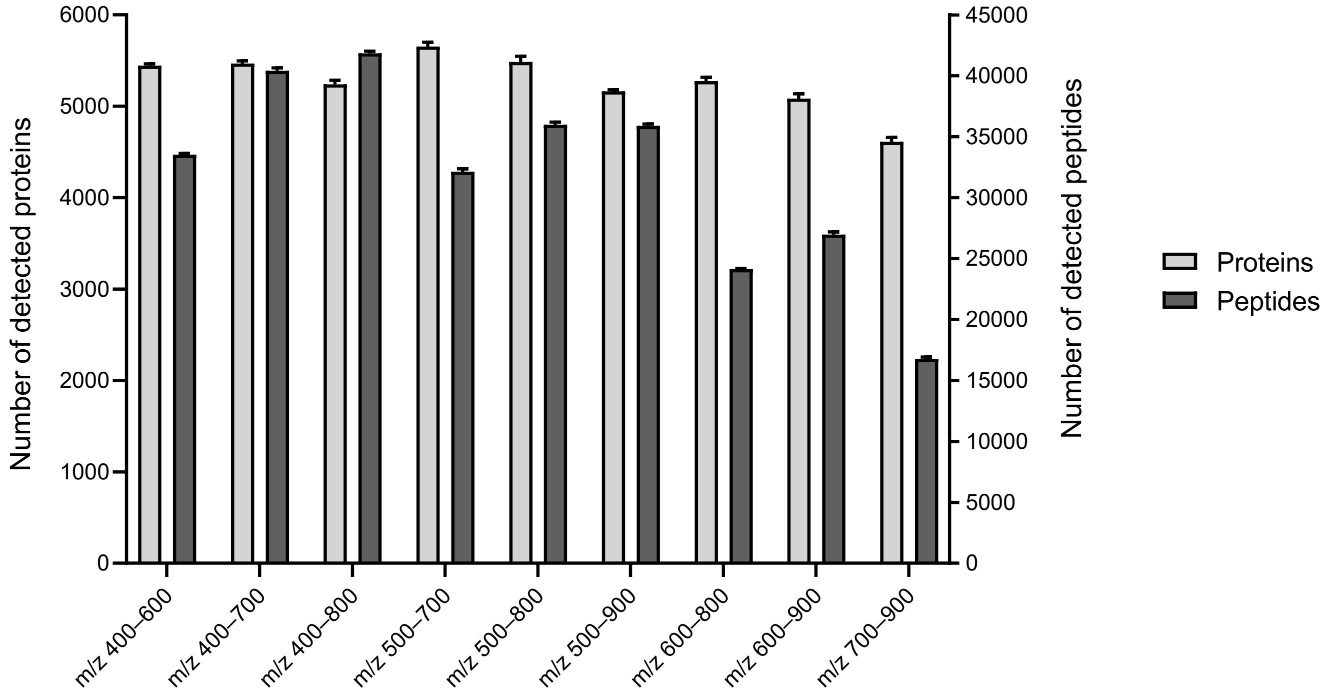

Figure S4. DIA-MS m/z range optimization for proteome analysis of auto-NANDA.

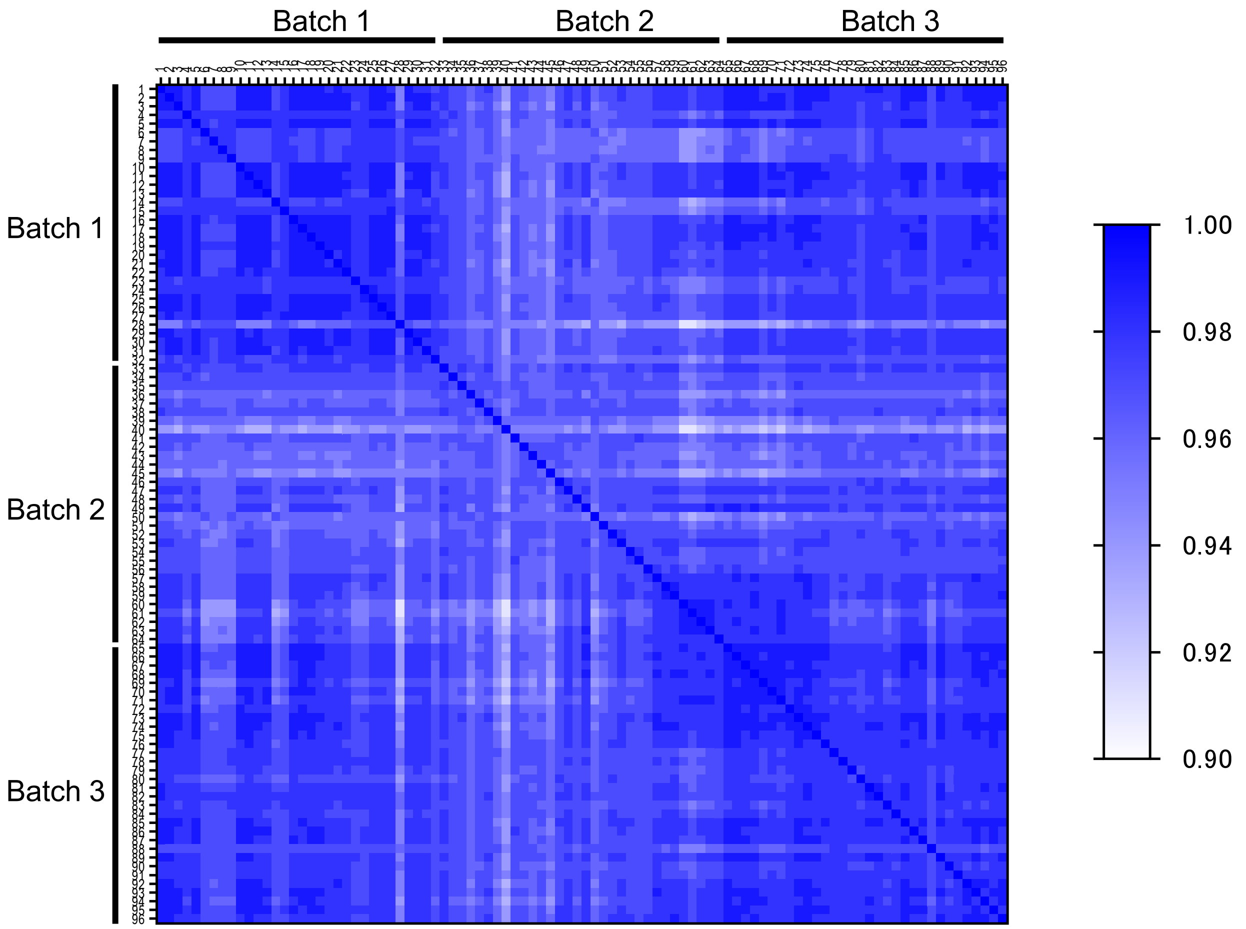

Figure S5. Reproducibility of data for 96 NANDA derived from three plates.
